# Hernia Treated by the Injection Method

**Published:** 1899-03

**Authors:** 


### HERNIA TREATED BY THE INJECTION METHOD.

Dr. W. W. Brown, of Blackstone, Mass., reports in the *Atlantic Medical Weekly*, cases treated by injection as follows:

℞ Creosote, *m* xv.

Morph. sulph , grs. ij.

Glycerite of tannin, ʒ j.

Witch hazel. ℥ iij.

Reduced by distillation to ℥ j.

M. Dose from v to x minims.

The mode of procedure, as practiced by me, is as follows:

Having placed the patient in a recumbent position and the hernia returned to the abdominal cavity, the left index finger is invaginated in the scrotum and the point of the finger pushed well into the canal. The syringe (I use a common hypodermic syringe, with a somewhat longer needle than the ordinary) is then taken in the right hand, using the left finger as a guide, the needle is thrust through the tissues, just beyond the tip of the finger, and slightly elevated toward the opening, and the fluid deposited. This is for oblique hernia; for the direct form, inject in the border or over external ring.

This operation should be repeated at intervals of from four to six days, six or eight times, or as often as the case may demand. After the injection, the parts should be slightly kneaded.—*Exchange.*

